# Understanding Viral dsRNA-Mediated Innate Immune Responses at the Cellular Level Using a Rainbow Trout Model

**DOI:** 10.3389/fimmu.2018.00829

**Published:** 2018-04-23

**Authors:** Sarah J. Poynter, Stephanie J. DeWitte-Orr

**Affiliations:** ^1^Department of Biology, University of Waterloo, Waterloo, ON, Canada; ^2^Department of Health Sciences, Wilfrid Laurier University, Waterloo, ON, Canada

**Keywords:** innate immunity, double-stranded RNA, type I interferon, antiviral, rainbow trout

## Abstract

Viruses across genome types produce long dsRNA molecules during replication [viral (v-) dsRNA]. dsRNA is a potent signaling molecule and inducer of type I interferon (IFN), leading to the production of interferon-stimulated genes (ISGs), and a protective antiviral state within the cell. Research on dsRNA-induced immune responses has relied heavily on a commercially available, and biologically irrelevant dsRNA, polyinosinic:polycytidylic acid (poly I:C). Alternatively, dsRNA can be produced by *in vitro* transcription (ivt-) dsRNA, with a defined sequence and length. We hypothesized that ivt-dsRNA, containing legitimate viral sequence and length, would be a more appropriate proxy for v-dsRNA, compared with poly I:C. This is the first study to investigate the effects of v-dsRNA on the innate antiviral response and to compare v-dsRNA to ivt-dsRNA-induced responses in fish cells, specifically rainbow trout. Previously, class A scavenger receptors (SR-As) were found to be surface receptors for poly I:C in rainbow trout cells. In this study, ivt-dsRNA binding was blocked by poly I:C and v-dsRNA, as well as SR-A competitive ligands, suggesting all three dsRNA molecules are recognized by SR-As. Downstream innate antiviral effects were determined by measuring IFN and ISG transcript levels using qRT-PCR and antiviral assays. Similar to what has been shown previously with ivt-dsRNA, v-dsRNA was able to induce IFN and ISG transcript production between 3 and 24 h, and its effects were length dependent (i.e., longer v-dsRNA produced a stronger response). Interestingly, when v-dsRNA and ivt-dsRNA were length and sequence matched both molecules induced statistically similar IFN and ISG transcript levels, which resulted in similar antiviral states against two aquatic viruses. To pursue sequence effects further, three ivt-dsRNA molecules of the same length but different sequences (including host and viral sequences) were tested for their ability to induce IFN/ISG transcripts and an antiviral state. All three induced responses similarly. This study is the first of its kind to look at the effects v-dsRNA in fish cells as well as to compare ivt-dsRNA to v-dsRNA, and suggests that ivt-dsRNA may be a good surrogate for v-dsRNA in the study of dsRNA-induced responses and potential future antiviral therapies.

## Introduction

Long dsRNA molecules (>40 bp) are immunomodulatory nucleic acids that can induce interferon (IFN) and an antiviral state across vertebrate species (1, 2). dsRNA is an important pathogen-associated molecule pattern (PAMP) produced by viruses; as demonstrated by the sheer number and diversity of receptors in the cytoplasm, endosome, and surface used by host cells to detect dsRNA (3). In vertebrates, dsRNA is a potent inducer of the type I IFN response, which produces a broad-spectrum antiviral state. Viral dsRNA is sensed in the cytoplasm by a wide range of receptors, such as retinoic acid-inducible gene-I (RIG-I), RNA helicase A/DHX9, and melanoma differentiation-associated protein 5 (MDA5), toll-like receptor 3 (TLR3) in the endosome, and class A scavenger receptors (SR-As) on the cell’s surface (1, 3, 4). When dsRNA is sensed in a cell it triggers a signaling cascade through various adaptor proteins, such as interferon-β promoter stimulator 1 or TIR-domain-containing adapter-inducing interferon-β (TRIF), activating transcription factors, such as interferon-regulator factor (IRF)3/7, and causing the production of IFN. IFNs are secreted from the cell and signal in an autocrine and paracrine fashion *via* their cognate receptor, interferon-α/β receptor, to initiate the Janus kinase and signal transducer and activator of transcription signaling pathway resulting in the expression of a group of genes containing an interferon-sensitive response element known cumulatively as interferon-stimulated genes [ISGs (5, 6)]. In rainbow trout (*Oncorhynchus mykiss*), there is an incredibly large repertoire of type I IFNs, as many as 22 members have been identified. Type I IFNs in fish have an intricate naming system, teleost type I IFNs are subdivided into group 1 or group 2 based on the number of cysteine residues and further into subgroups (a–f) based on phylogenetic analysis (7). These naming systems have no relation to the alpha or beta system used in mammals (7). IFN1 is a type I IFN, belonging to group I and subgroup a (7) and is used in this study as a representative transcript indicative of type I IFN expression. IFNs in fish stimulate expression of a panel of ISGs including molecules from the IFN signaling pathway such as IRF3/7 and antiviral effectors that limit viral infection, including Myxovirus resistance 1 (Mx1), viral hemorrhagic septicemia virus (VHSV)-induced gene (vig)-1, vig-3, and vig-4 (6, 8, 9). vig-4 is a VHSV-induced gene; the deduced protein contains tetratricopeptide repeat motifs and shows similarities to the ISG56/IFIT1 family of ISGs (9, 10). This study chose vig-4 as a representative transcript indicative of ISG expression because it has been used as a representative ISG in previous studies (11–14) and is upregulated more strongly than other ISGs, such as Mx1 (15). The dsRNA-induced accumulation of ISG proteins produces a protective antiviral state in rainbow trout cells against various viruses (15, 16).

DsRNA sensed by a host cell in a natural system would be produced by viral infection, described here as v-dsRNA. As viruses replicate, dsRNA is produced as a by-product of replication, a genomic fragment, or transcribed from DNA by host proteins (17–20). The potent immune stimulatory nature of dsRNA makes it a candidate molecule for antiviral therapies and vaccine adjuvants, as well as for use in type I IFN studies. Unfortunately, v-dsRNA is difficult to collect from viruses in quantities useful for experimental scenarios and likely impossible for industrial applications. In the late 1960s, a synthetic form of dsRNA, polyinosinic:polycytidylic acid (poly I:C), was identified as a potent IFN-inducer and was considered a “viral mimic” (21–23). Poly I:C is clearly different from dsRNA produced by a virus; poly I:C lacks sequence variation and natural structures, contains a range of lengths and one strand contains exclusively a modified inosine nucleotide (24). Owing to these differences, poly I:C is not sensed the same nor does it induce responses exactly the same as *in vitro* transcribed (ivt-) dsRNA (24–27). In plasmacytoid dendritic cells only ivt-dsRNA was able to stimulate IFN-α production, poly I:C did not (24). In rainbow trout cells, ivt-dsRNA induced a faster, stronger IFN1 and IFN2 response compared with poly I:C even when poly I:C was of much longer lengths (15). In addition, in mice, poly I:C is recognized by MDA5 whereas ivt-dsRNA and v-dsRNA activated RIG-I (25, 28). For TLR3, human TLR3 but not teleost TLR3 has a much higher affinity for poly I:C than ivt-dsRNA (25, 29).

The current state of research regarding responses to dsRNA largely relies on the use of poly I:C; however, studies of individual receptors are shifting toward ivt-dsRNA, likely for the ease of controlling length (25, 27, 30). Length has been shown to influence the magnitude of immune response in cells and dsRNA receptor types show length requirements and specificities (15, 25, 27, 29). For example, longer dsRNA molecules have been shown to induce a strong IFN response (27), and RIG-I has been shown to sense dsRNA molecules under 1,000 bp in length, while MDA5 senses lengths greater than 1,000 bp (28, 30). The effect of dsRNA sequence on IFN induction is also poorly studied; there were no detectable sequence motifs for MDA5 activation identified from vaccinia virus-derived dsRNA (31). One example of sequence dependence is the cytoplasmic dsRNA receptor oligoadenylate synthetase (OAS) that requires a 4 bp-specific motif for binding (32). Few studies have looked at the antiviral response induced by v-dsRNA, and any studies that do exist have all used mammalian models. Specifically, v-dsRNA derived from encephalomyocarditis virus, vaccinia virus, and reovirus induced potent IFN responses in Vero, HeLa, and murine embryonic fibroblasts, respectively (28, 31). ivt-dsRNA is an alternative source of synthetic dsRNA that retains some features of v-dsRNA and can be produced on a larger scale. To the best of our knowledge, there have been no studies directly comparing a v-dsRNA and an ivt-dsRNA molecule of matched length and sequence, therefore it is unknown if ivt-dsRNA induces a comparable immune response to v-dsRNA.

Rainbow trout were used in this study as a model fish species for their importance in aquaculture and the existing knowledge base of the rainbow trout type I IFN and antiviral response (15, 33). In rainbow trout cell lines, ivt-dsRNA or poly I:C induces type I IFN and an antiviral state and similarly whole rainbow trout pretreated with poly I:C also showed decreased susceptibility to a fish virus (15, 34). Three aquatic viruses were used in this study: chum salmon reovirus (CSV), which has a segmented dsRNA genome that consists of 11 segments between 3,947 and 783 bp (35), infectious pancreatic necrosis virus (IPNV), which is a non-enveloped *Aquabirnavirus* with a bisegmented dsRNA genome (36) and VHSV, which is an enveloped rhabdovirus with a negative-sense ssRNA genome (37). All three viruses readily infect rainbow trout cells, including RTG-2, a rainbow trout gonadal cell line (38, 39). Mammalian reoviruses have previously been used as a source of dsRNA of different lengths and total genomic dsRNA has been used as an immune stimulus (14, 28, 40). VHSV and IPNV both represent important disease in the fish aquaculture industry and ecology as they have wide host ranges and can cause large die-offs of fish (36, 37, 41).

This study compares IFN-mediated responses induced by three forms of dsRNA: v-dsRNA, ivt-dsRNA, and poly I:C. The dsRNA is this study was delivered extracellularly. In a viral infection, the dsRNA would be intracellular during its production and released to the extracellular space in the case of cell lysis where it could be recognized by neighboring cells. In the case of a dsRNA-based therapy the dsRNA would be delivered to the cell surface and not to the cytoplasm. Because the dsRNA was delivered extracellularly, the surface receptor for these dsRNA molecules was investigated. The ability of v-dsRNA from aquatic viruses to induce IFNs, ISGs, and an antiviral response was quantified. Length- and sequence-matched v-dsRNA and ivt-dsRNA were compared with poly I:C for their ability to induce IFNs, ISGs, and mount an antiviral state against IPNV and VHSV. The results from this study provide valuable insight with regards to how fish cells respond to viral dsRNA as opposed to poly I:C, with applications for novel dsRNA-based therapies.

## Materials and Methods

### Cell Culture and Virus Propagation

Two rainbow trout cell lines were used in this study to measure dsRNA-mediated responses: RTG-2, derived from rainbow trout gonad (42) and RTgutGC, derived from rainbow trout intestine (43). Epithelioma papulosum cyprinid (EPC) and Chinook salmon embryonic cell line (CHSE-214) were used for viral propagation. All cell lines used in this study were obtained from N. Bols (University of Waterloo, Waterloo, ON, Canada). All cell lines were grown in 75 cm^2^ plastic tissue culture flasks (BD Falcon, Bedford, MA, USA) at room temperature in Leibovitz’s L-15 media (HyClone, Logan, UT, USA) supplemented with 10% v/v fetal bovine serum (FBS; Fisher Scientific, Fair Lawn, NJ, USA) and 1% v/v penicillin/streptomycin (P/S) (10 mg/mL streptomycin and 10,000 U/mL penicillin; Fisher Scientific). All dsRNA treatments were delivered extracellularly by addition of dsRNA to cell culture media.

### Virus Propagation

Viral hemorrhagic septicemia virus-IVb (strain U13653) was propagated on monolayers of EPC (44) cells; CSV and IPNV were propagated on CHSE-214; and all viruses were propagated at 17°C (45). Virus containing media [L-15 with 2% v/v FBS (Fisher Scientific)] was collected 4–7 days post-infection, filtered through a 0.45 µm filter (Nalgene, Rochester, NY, USA) and kept frozen at −80°C. The 50% tissue culture infective dose (TCID_50_)/mL values were estimated according to the Reed and Muench method (46). The origin of the viruses used in this study has been described previously (47).

### Polyinosinic:Polycytidylic Acid

High-molecular weight (HMW) poly I:C (InvivoGen, San Diego, CA, USA) stocks were prepared at 1 mg/mL, and low-molecular weight (LMW) poly I:C (InvivoGen) stocks were prepared at 10 mg/mL, both were diluted in phosphate-buffered saline (PBS) (HyClone), and aliquots were stored at −20°C. Before use, aliquots were heated to 55°C for 15 min and then allowed to cool to room temperature for 20 min.

### Synthesis of *In Vitro* Molecules

*In vitro* transcribed dsRNA molecules were produced as previously described using the MegaScript RNAi kit [Fisher Scientific (15)]. All primers used for synthesizing dsRNA, Table 1, had the T7 promoter sequence added to the 5′ end, TAATACGACTCACTATAGGGAG. The following molecules were prepared: the full-length CSV segment 6 (2,052 bp); a 300 bp internal segment of CSV segment 1 (CSVseg1); a 200 bp segment of rainbow trout GAPDH, rainbow trout Mx3, and an internal segment of the VHSV G gene (3,851–4,048 bp) that has been previously described (15). Where needed, nucleotide distribution was calculated using Genomatix: DNA sequence toolbox.^1^

**Table 1:** Primers used for *in vitro* transcription of dsRNA and qRT-PCR.

Target	5′–3′	Length (bp)	Ta	Accession number
**dsRNA**				
GAPDH (seq1)	F—TGGCATCTCCTTCAACGACAA R—GCTGGGGGTACTATGGGTGT	200	55	NM_001124246.1
Mx3 (seq2)	F—AGGACTCGGCAGAAAGGATA R—TTCTCCCTCGATCCTCTGGT	200	55	U47946.1
V200 (seq3)	F—TCAGATGAGGGGAGCCACA R—CGCATGATCTGGCCATCAA	200	52	(15)
CSV seg1	F—TATGGTCCCCACGTCCTGAT R—GCCTCCTACGTCACTCATCG	300	50	AF418294.1
CSV seg6	F—TATCTCCTTGCGCCCTTCTC R—AATAGTCATCCCCCTCCGGC	2,052	60	AF418299.1

**qRT-PCR**				
IFN1	F—AAAACTGTTTGATGGGAATATGAAA R—CGTTTCAGTCTCCTCTCAGGTT	141	55	(48)
vig-4	F—GGGCTATGCCATTGTCCTGT R—AAGCTTCAGGGCTAGGAGGA	151	55	(15)
β-Actin	F—GTCACCAACTGGGACGACAT R—GTACATGGCAGGGGTGTTGA	174	55	(49)

*Forward (F) and reverse (R) primer sequences (5′–3′), length of product (bp), annealing temperature (Ta), and accession number of source sequence are provided. For dsRNA, the length of the final dRNA product is provided*.

### Extraction of v-dsRNA and Isolation of Segments

To extract the CSV viral genome, CSV was propagated as described earlier; after complete destruction of the monolayer, the virus-containing media was collected, and cell debris was pelleted by centrifugation at 3,000 × *g* for 5 min. The supernatant was mixed with poly ethylene glycol BioUltra 8000 to a final concentration of 10% w/v (Sigma-Aldrich, St. Louis, MO, USA; catalog number: 89510) and sodium chloride to a final concentration of 0.6% w/v and mixed on a Corning LSE Digital Microplate Shaker at 1,400 RPM 4°C overnight [Corning, Tewksbury, MA, USA (50)]. The solution was then centrifuged at 17,000 × *g* in a Sorvall Legend Micro 17 microcentrifuge for 20 min (Fisher Scientific). The resulting pellet was resuspended in 100 µL PBS overnight at 4°C, and the RNA was extracted using TRIzol reagent (Invitrogen, Carlsbad, CA, USA) as per the manufacturers’ instructions. To isolate segments of the genome, total genomic CSV dsRNA was run on a 1% agarose TAE gel containing GelGreen (1:10,000 dilution; Biotium Inc., Fremont, CA, USA). The band of interest was cut out and purified using the QIAquick gel extraction kit (Qiagen, Hilden, Germany) and quantified using a NanoDrop Lite Spectrophotometer (Thermo Fisher Scientific). The v-dsRNA preparation was validated as pure dsRNA by two methods: acridine orange stained gel to confirm only red stained nucleic acids were visible, and differential nuclease degradation using RNase A and RNaseIII to ensure degradation by RNaseIII alone. These methods for visualizing dsRNA have been described previously (51). 20 ng of resulting products was re-quantified by gel densitometry to confirm the NanoDrop readings. The matched ivt-dsRNA molecule was also gel purified and quantified as above for consistency.

### Labeling of dsRNA

The CSV seg1 ivt-dsRNA was labeled using the Ulysis Alexa Fluor 546 Nucleic Acid Labeling Kit (Fisher Scientific) as previously described (15). Unbound fluorophores were removed using a BioRad p30 spin column (BioRad, Hercules, CA, USA).

### Competitive Binding Assay

RTgutGC cells were seeded at a density of 1 × 10^5^ cells/well on glass coverslips in a 12-well tissue culture plate. After attaching overnight cells were pretreated with 200 µL of L-15 containing LMW, HMW, poly I, or poly C at 100 µg/mL, total native dsRNA at 40 µg/mL, or control L-15 alone for 30 min. After this incubation, 1.25 µg (5 µg/mL) of labeled CSVseg1 dsRNA was added to the well with 50 µg/mL of DEAE-dextran. DEAE-dextran is used in this context as a method of ensuring dsRNA reaches the cells in quantities sufficient for detection by fluorescence microscopy (1). Six hours posttreatment cells were washed 3× with PBS, fixed for 10 min with 10% neutral buffered formalin (Fisher Scientific), nuclei were counterstained with 10 µg/mL 4′,6-diamidino-2-phenylindole (DAPI; Fisher Scientific) and mounted on coverslips with SlowFade Gold mounting medium (Fisher Scientific) for visualization. Images were captured using an inverted fluorescence microscope (Nikon Eclipse TiE with Qi1 camera) and analyzed using Nikon NIS elements. A blinded third-party researcher performed the fluorescence quantification measurements. Three images were captured of each treatment for each independent replicate. Alexa Fluor 546 fluorescence intensity was measured by automatic selection of the area surrounding DAPI stained nuclei, total number of cells within the image were counted, and the intensity/cell was calculated. Intensity is presented as percentage of the *in vitro* only control with no pretreatment.

### Cell Protection Assays

RTG-2 cells were seeded at 1 × 10^4^ cells/well in 96-well plates and allowed to attach overnight in regular growth media. For each condition, there were triplicate wells, and for each assay there was an untreated/uninfected control and an untreated/infected control. Cells were treated with the indicated concentration of dsRNA for 3 or 6 h at 20°C in 50 µL of media containing L-15, 1% P/S, and 2% FBS. When dsRNA concentration was 0.01 nM, 6 h was selected to ensure a productive antiviral state before infection; 3 h was found to be insufficient (data not shown). After dsRNA pretreatment, the virus was added directly to the media to produce the necessary multiplicity of infection (MOI) (10 for VHSV, 0.2 or 0.02 for IPNV). Cells were incubated at 17°C for 4–7 days until desired accumulation of cytopathic effect occurred. Cells were rinsed 2× with PBS and a 5% v/v alamarBlue solution in PBS was added (Invitrogen). Cells were incubated at room temperature for 1 h in the dark, and fluorescence was measured using a Synergy HT plate reader (BioTek, Winooski, VT, USA). Data are presented as percentage of control, uninfected cells.

### RNA Extraction, cDNA Synthesis, and qRT-PCR

5 × 10^5^ cells/well of RTG-2 cells were plated in a 6-well plate and allowed to attach overnight. Media were removed, and cells were treated with dsRNA at the noted concentration in full-growth media, or with media only. After the indicated incubation time, media were removed, and RNA extracted with TRIzol as per the manufacturers’ instructions (Thermo Fisher Scientific). Possible contaminating DNA was removed using the TURBO DNA-free kit (Fisher Scientific). cDNA was synthesized using iSCRIPT (BioRad), and 1 µg of RNA. qRT-PCR has been previously described for the primers used in this study (15). A no-reverse transcriptase (NRT) control was used to identify genomic contamination, a peak earlier than 35 cycles in the NRT control was considered excessive DNA contamination, no samples showed a peak earlier than this threshold. Melting curve analysis was completed (65–95°C with a read every 5 s) to determine primer specificity, only single peaks were produced. In addition to the NRT control, primers were designed to span introns for both β-actin and IFN1; as such genomic contamination would produce a much larger product, which would be evident in the melting curve analysis. This technique could not be performed for vig-4, as it is predicted to be intron-less. In addition, there are two predicted copies of vig-4 in the rainbow trout genome, the primers used in this study would amplify both variants; however, all sequencing performed identified only the published vig-4 sequence (NM_001124333.1).

### Statistical Analysis

All data presented were derived from at least three independent experiments. Data were graphed and statistically analyzed using GraphPad Prism version 7.00 for Windows, GraphPad Software, La Jolla, CA, USA.^2^ Statistical analyses were completed using a one- or two-way ANOVA with Tukey multiple comparison test, alpha = 0.05, *P* value < 0.05 considered significant; qRT-PCR data were log2 transformed before analysis. Data points labelled with the same letter do not have a statistically different average.

## Results

### dsRNA From Different Sources Bind to the Same Surface Receptor

**Figure 1:**
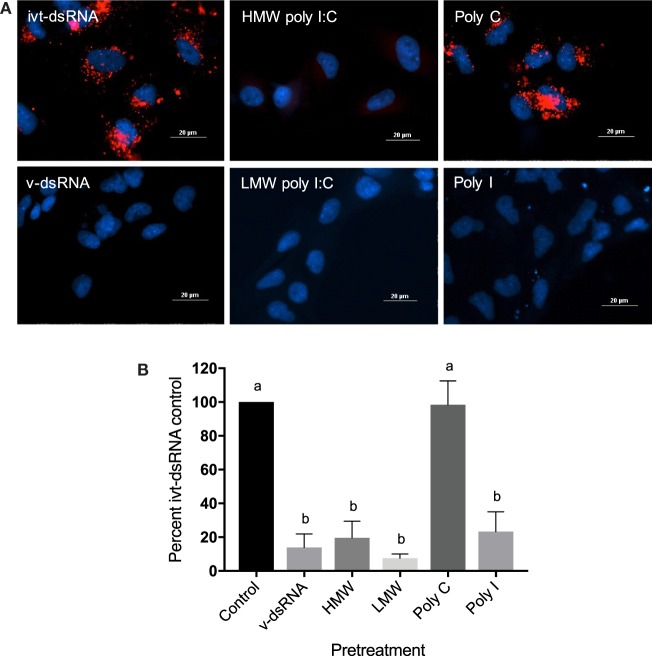
*In vitro* transcribed dsRNA, viral dsRNA, and polyinosinic:polycytidylic acid (poly I:C) bind the same class A scavenger receptor in RTgutGC cells. RTgutGC cells were pretreated for 3 h with high-molecular weight (HMW) poly I:C, low-molecular weight (LMW) poly I:C, poly I, or poly C at 100 µg/mL, or total chum salmon reovirus genome (v-dsRNA) at 40 µg/mL, or control media. Cells then received 1.25 µg (5 µg/mL) of Alexa Fluor 546 labeled CSVseg1 dsRNA (ivt-dsRNA) with 50 µg/mL of DEAE-dextran. 6 h posttreatment cells were formalin fixed, and nuclei were counterstained with diamidino-2-phenylindole; slides were visualized using an inverted fluorescence microscope (Nikon Eclipse TiE with Qi1 camera). **(A)** Representative images of cells with or without pretreatment, dsRNA = red, nuclei = blue. **(B)** Fluorescence intensity per cell was quantified using Nikon NIS-elements software and is presented as percent of the ivt-dsRNA only control, no pretreatment. Data represent three independent replicates and were analyzed statistically by one-way ANOVA, alpha = 0.05; a *P* value < 0.05 considered significant.

RTgutGC cells, previously shown to bind poly I:C by SR-As (49), were treated with fluorescently labeled ivt-dsRNA, and punctate cell-associated binding was observed (Figure 1A). Cells were then pretreated with an excess of poly I:C, either high molecular weight (HMW) or low molecular weight (LMW), total CSV v-dsRNA, poly I or poly C (Figures 1A,B). Both sizes of poly I:C and the total v-dsRNA significantly blocked binding of the labeled ivt-dsRNA (Figure 1B). poly I is a competitive ligand for SR-A binding whereas poly C is a molecule of similar chemical structure that is non-competitive for SR-As. poly I significantly blocked ivt-dsRNA binding while poly C did not (Figure 1B).

### v-dsRNA Elicits a Length-Dependent, Protective Type I IFN Response

**Figure 2:**
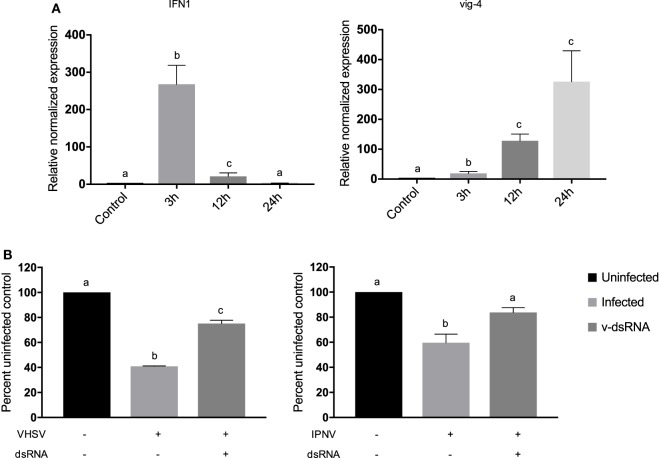
Viral dsRNA induces IFN1 and vig-4 transcripts over time and induces a protective antiviral state against viral hemorrhagic septicemia virus (VHSV) and infectious pancreatic necrosis virus (IPNV) in RTG-2 cells. **(A)** RTG-2 cells were stimulated with 10 ng/mL of total chum salmon reovirus dsRNA (v-dsRNA); IFN1 and vig-4 transcripts were measured by qRT-PCR at 3, 12, and 24 h, normalized to β-actin, and presented as values relative to an unstimulated control. **(B)** RTG-2 cells were pretreated with 10 ng/mL v-dsRNA for 3 h and then infected with VHSV at a multiplicity of infection (MOI) of 10 or IPNV at an MOI of 0.2. After 4–7 days, a fluorescent indicator dye, alamarBlue, was used to measure cell viability, and data are presented as the percentage of an untreated, uninfected control. Data represent three independent replicates and were analyzed statistically by one-way ANOVA, alpha = 0.05; a *P* value < 0.05 considered significant.

RTG-2 cells were treated with 10 ng/mL of total CSV v-dsRNA, and significant induction of an IFN (IFN1) and an ISG (vig-4) was observed at the transcript level using qRT-PCR at 24 h posttreatment (Figure 2A). IFN1 transcript production peaked at 3 h and was no longer measurable by 24 h and vig-4 production peaked at 24 h (Figure 2A). Previously, concentrations lower than 10 ng/mL of ivt-dsRNA have been shown to induce IFN and ISG transcripts in RTG-2 cells (15). To test the ability of v-dsRNA to establish a functional antiviral state, RTG-2 cells were pretreated for 3 h with 10 ng/mL of total v-dsRNA and then infected with VHSV (MOI: 10) or IPNV (MOI: 0.2; Figure 2B). There was significant protection observed against both viruses when cell viability was measured using alamarBlue.

**Figure 3:**
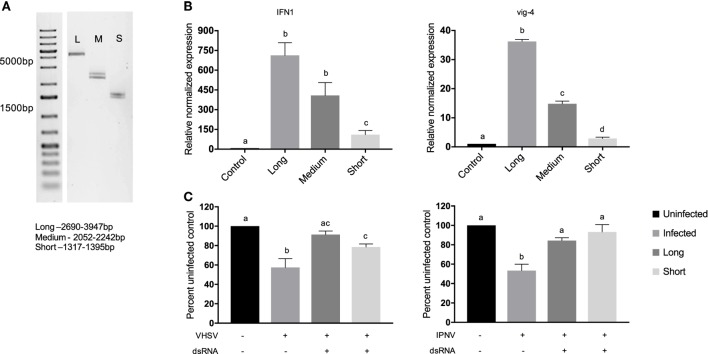
Long v-dsRNA segments induces higher levels of IFN1 and vig-4 transcripts but similar antiviral responses against viral hemorrhagic septicemia virus (VHSV) or infectious pancreatic necrosis virus (IPNV) in RTG-2 cells. Segments of the chum salmon reovirus genome were isolated based on length, segments fell into long (2,690–3,947 bp), medium (2,052–2,242 bp), or short (1,317–1,395 bp) segment groups. **(A)** 20 ng of the isolated segments was run on a 1% agarose gel stained with ethidium bromide for visualization. **(B)** RTG-2 cells were treated with 0.05 nM dsRNA for 3 h, and IFN1 and vig-4 transcripts were measured by qRT-PCR, normalized to β-actin, and presented as values relative to an unstimulated control. **(C)** RTG-2 cells were pretreated with 0.05 nM dsRNA for 3 h and then infected with VHSV at a multiplicity of infection (MOI) of 10 or IPNV at an MOI of 0.2. After 4–7 days a fluorescent indicator dye, alamarBlue, was used to measure cell viability, and data are presented as the percentage of an untreated, uninfected control. Data represent at least three independent replicates and were analyzed statistically by one-way ANOVA, alpha = 0.05; a *P* value < 0.05 considered significant.

To look at the effects of v-dsRNA length on the immune response, portions of the CSV genome grouped as large, medium, and small segments were isolated, and RTG-2 cells were treated with 0.05 nM of each molecule for 3 h (Figures 3A,B). The concentration used was chosen because in previous studies this concentration demonstrated length effect differences between ivt-dsRNA molecules. In addition, molar amounts were used to measure the effect of length as opposed to number of molecules (15). It should be noted that these v-dsRNA molecules are different sequences as well as lengths. There was a length-dependent response seen in the RTG-2 production of IFN1 and vig-4 transcripts, with the long segments inducing significantly more IFN1 and vig-4 transcripts than the short segments. For both IFN1 and vig-4, the medium segment fell in the middle range of long and short, and there was a significant difference between long and medium-induced vig-4 production. A corresponding antiviral assay against VHSV and IPNV was completed similarly to the total v-dsRNA assay described earlier (Figure 3C). While both long and short v-dsRNA protected cells significantly from viral infection, there were no significant differences between the two lengths.

### Length- and Sequence-Matched v-dsRNA and ivt-dsRNA Induce IFNs and an Antiviral State Similarly

**Figure 4:**
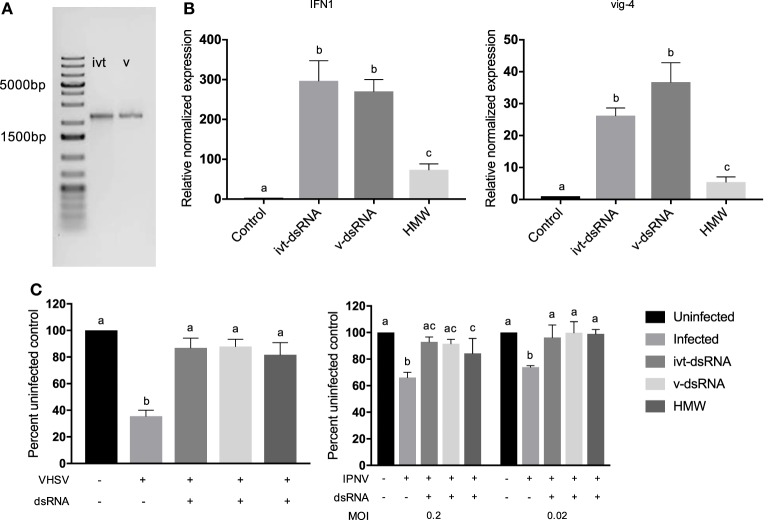
Length- and sequence-matched ivt- and v-dsRNA induced similar levels of IFN1 and vig-4 transcripts and similar antiviral state against viral hemorrhagic septicemia virus (VHSV) and infectious pancreatic necrosis virus (IPNV) infections in RTG-2 cells. A segment of the chum salmon reovirus genome (segment 6, 2,052 bp) was isolated (v-dsRNA), and a length- and sequence-matched dsRNA molecule was transcribed *in vitro* (ivt-dsRNA). High-molecular weight (HMW) polyinosinic:polycytidylic acid was included as a treatment control. **(A)** 20 ng of the isolated segments was run on a 1% agarose gel stained with ethidium bromide for visualization. **(B)** RTG-2 cells were treated with 0.01 nM dsRNA for 3 h, and IFN1 and vig-4 transcripts were measured by qRT-PCR, normalized to β-actin, and presented as values relative to an unstimulated control. **(C)** RTG-2 cells were pretreated with 0.01 nM dsRNA for 6 h and then infected with VHSV at a multiplicity of infection (MOI) of 10 or IPNV at an MOI of 0.2 or 0.02. After 4–7 days, a fluorescent indicator dye, alamarBlue, was used to measure cell viability, and data are presented as the percentage of an untreated, uninfected control. Data represent least three independent replicates and were analyzed statistically by one-way ANOVA **(B)** or two-way ANOVA **(C)**, alpha = 0.05; a *P* value < 0.05 considered significant.

To compare the IFN response induced by v-dsRNA and ivt-dsRNA directly, v-dsRNA from the CSV genome (segment 6, 2,052 bp) was isolated, and a matching ivt-dsRNA molecule of the same length and sequence was synthesized to match (Figure 4A). Cells were stimulated with 0.01 nM of v-dsRNA or ivt-dsRNA, respectively. This is a lower molar amount of dsRNA than other assays in this study, due to the difficulty of isolating usable quantities of a single v-dsRNA segment. There was significant induction of both IFN1 and vig-4 transcripts by both molecules, and no significant difference was observed between the ivt-dsRNA and v-dsRNA molecules (Figure 4B). This trend continued with the antiviral assays, where both ivt-dsRNA and v-dsRNA protected cells against IPNV and VHSV-induced cell death, but there were no significant differences in the protection between the two (Figure 4C).

For comparison purposes, a 0.01 nM HMW poly I:C (average length 3,000 bp) control was included for both qRT-PCR and antiviral assays. There was significantly less induction of IFN1 and vig-4 transcript production from the HMW poly I:C at the 3 h time point (Figure 4B). With VHSV infection, all three dsRNA molecules protected similarly; however, with IPNV at an MOI of 0.2 ivt-dsRNA and v-dsRNA provided complete protection (with viability levels similar to uninfected control cells) but poly I:C did not. By an MOI of 0.02, all three dsRNA molecules provided the same amount of protection (Figure 4C).

### Molecules of the Same Length but Different Sequence Had Similar Effects

**Figure 5:**
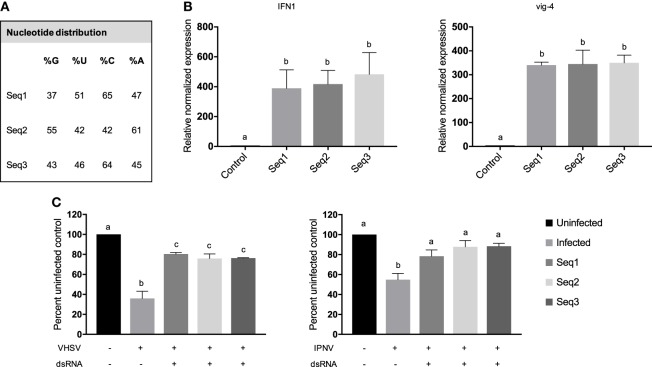
ivt*-*dsRNA of the same length and different sequence induced similar levels of IFN1 and vig-4 transcripts and protective antiviral states against viral hemorrhagic septicemia virus (VHSV) and infectious pancreatic necrosis virus (IPNV) in RTG-2 cells. Three *in vitro* transcribed dsRNA molecules of the same length, but different source sequences were used to test the effects of sequence on innate immune response. **(A)** Genomatix was used to calculate the nucleotide distribution within the three sequences, and the percentage of each nucleotide is shown. **(B)** RTG-2 cells were treated with 0.05 nM dsRNA for 6 h, and IFN1 and vig-4 transcripts were measured by qRT-PCR, normalized to β-actin, and presented as values relative to an unstimulated control. **(C)** RTG-2 cells were pretreated with 0.05 nM dsRNA for 3 h and then infected with VHSV at a multiplicity of infection (MOI) of 10 or IPNV at an MOI of 0.2. After 4–7 days, a fluorescent indicator dye, alamarBlue, was used to measure cell viability, and data are presented as the percentage of an untreated, uninfected control. Data represent three independent replicates and were analyzed statistically by one-way ANOVA, alpha = 0.05; a *P* value < 0.05 considered significant.

To test the effect of sequence on the magnitude of the immune response by RTG-2 cells, three length-matched ivt-dsRNAs were made with different sequences, including different source material (two rainbow trout genes and one viral gene), and different nucleotide composition (Figure 5A). IFN1 and vig-4 transcripts were significantly induced following treatment with 0.05 nM dsRNA for 3 h, but no significant differences between the three sequences were detected (Figure 5B). This matched the antiviral assays for both VHSV and IPNV, where protection was seen but no significant differences between molecules (Figure 5C).

## Discussion

While poly I:C is an attractive dsRNA to use in studies of the dsRNA-mediated immune response, it is not biologically relevant and as such is either too potent (21) or not potent enough (52) when modulating the innate immune response. We hypothesized that ivt-dsRNA would be a better molecule for modulating innate antiviral immune responses, as it has natural sequence variation and defined length. The goal of this study was to test whether ivt-dsRNA was a comparable surrogate for v-dsRNA and to better understand v-dsRNA and ivt-dsRNA-mediated innate antiviral immune responses using a rainbow trout cell model. This is the first study to investigate v-dsRNA and its effects in an aquatic vertebrate system. The use of the two difference cell lines helped provide answers to two separate questions, (1) whether the molecules are bound by a common scavenger receptor and (2) whether the molecules were inducing similar antiviral responses. To answer the first point, RTgutGC was used, as this cell line has been shown previously to express functional SR-As, surface receptors for dsRNA (49). RTG-2 was used to answer the second point as it has previously been shown to respond to low concentrations of dsRNA and is permissive to infection by both aquatic viruses used to test the establishment of an antiviral state (15, 16, 38).

The dsRNA in this study was delivered extracellularly; they were added directly to the media instead of being transfected into the cell. In a viral infection, dsRNA would be intracellular during its production and released to the extracellular space during a lytic infection where it could be recognized by neighboring cells. In the case of a dsRNA-based therapy, the dsRNA would be delivered to the cell surface and not to the cytoplasm. If dsRNA-based therapies are viable, they need to be able to bind to the cell’s surface, thus the mechanism of recognition of ivt-dsRNA compared with v-dsRNA at the cell surface is important to discern. Poly I:C (both LMW and HMW) and v-dsRNA effectively blocked the binding of labeled ivt-dsRNA molecules in RTG-2 cells, suggesting that all three dsRNA molecules do indeed bind SR-As on the cell surface. This is consistent with previous studies in mammals that have shown ivt-dsRNA, poly I:C, and viral dsRNA are bound by SR-As (1, 53–56). A study in mouse splenocytes found HMW poly I:C but not LMW was bound by SR-As, and therefore both LMW and HMW poly I:C were tested in this study for their ability to block binding (54). In this study, no differences were observed between LMW and HMW poly I:C’s ability to block ivt-dsRNA binding, suggesting SR-A binding capabilities to dsRNA in mice differ from rainbow trout.

Next, the ability of v-dsRNA to induce the IFN pathway and antiviral response was tested. v-dsRNA induced IFN1 and vig-4 transcripts with similar kinetics to what was previously reported with ivt-dsRNA (49) in that IFN1 transcript peaked at 3 h and the ISG vig-4 accumulated over time. This antiviral response effectively reduced virus-induced cell death for two important fish pathogens, VHSV-IVb and IPNV. This is the first study in fish to demonstrate v-dsRNA as a stimulant of type I IFN and an inducer of an antiviral state against aquatic viruses.

This is not, however, the first study to use the reovirus genome as a source of v-dsRNA. Previously, the total reovirus genome as well as isolated segments has been used as immune-inducing molecules in mammalian cells (14, 28, 40). One of the first studies using v-dsRNA as an immunostimulant was in 1967 when an isolated reovirus genome was injected into rabbits and induced IFN (57). The cellular response to the reovirus genome has been demonstrated in mouse embryonic fibroblasts and human embryonic kidney cells (HEK293), both cell types producing IFN-β following v-dsRNA treatment (14, 28). Reoviruses are used for these types of studies due to the relative abundance of dsRNA produced during infection. Current studies are underway to optimize methods to isolate dsRNA from viruses of other genome types.

Similar to ivt-dsRNA, v-dsRNA induced IFN pathways, and in some cases an antiviral state, in a length-dependent manner. The long v-dsRNA molecule induced more IFN1 than short and long v-dsRNA induced more vig-4 transcript than medium and short. At the level of an antiviral state, there were no differences between long and short v-dsRNA induced protection for either VHSV or IPNV infection; however, long v-dsRNA did appear to provide protection similar to control in the VHSV infection model while the short molecule did not (Figure 3C). These differences are indeed subtle, which is unsurprising as the long segment is only 2× the length of the short, whereas previous studies of length have shown an effect with molecules with a 6× and 10× difference in length in RTG-2 cells (15). It can then be hypothesized if shorter v-dsRNA molecules were used a greater difference in antiviral assays would be observed. It should be noted that while the molecules are of different lengths they are also of different sequences, and this could be a confounding variable in this study. While future studies may address this issue by digesting native dsRNA genome fragments or ligating fragments together, this study aimed to focus on native molecules that were as unmodified as possible. Even so, evidence from this study and previous work suggest sequence does not play a role in levels of IFN induction (31, 58).

Next, a direct comparison between length- and sequence-matched v-dsRNA and ivt-dsRNA was performed, and no significant differences were observed between the immune gene transcript induction and antiviral state established by v-dsRNA and the matched ivt-dsRNA. HMW poly I:C, however, even with a much longer average length, was not as effective at inducing IFNs or ISGs and required a lower MOI of IPNV to protect cells similarly to v- or ivt-dsRNA. These data suggest that indeed ivt-dsRNA but not poly I:C could be used as a surrogate for v-dsRNA. Indeed, previous work has shown that poly I:C induced IFN and ISG kinetics differently from ivt-dsRNA in rainbow trout cells (15). These differences continue between fish and humans with regard to poly I:C. Human TLR3 responds very strongly to poly I:C, whereas this was not the case for a fish (*Takifugu rubripes*) TLR3, which responded most strongly to one length of ivt-dsRNA compared with poly IC or other lengths of ivt-dsRNA (29). *In vivo*, poly I:C has had mixed effects as an antiviral therapy, being an effective protection mechanism against red-spotted grouper necrosis virus in sevenband grouper (59), but in zebrafish (*Danio rerio*) infected with VHSV poly I:C was able to delay symptoms but only prevented mortality in 5% of fish (60). Clearly, there is room for increasing the efficacy of dsRNA-based antiviral therapies in fish past the protection that poly IC can provide.

It should be noted that there are differences between v-dsRNA generated *in situ* with the v-dsRNA isolated for this study. One difference is the lack of dsRNA-associated proteins in the extracted v-dsRNA. Viruses use many mechanisms to hide dsRNA, one method is the production of proteins that bind dsRNA and effectively hide it from host receptors (61). Host cells also have a number of dsRNA-binding proteins that could modify dsRNA availability and potency (21). Extracting v-dsRNA through a column or phenol/chloroform extraction would remove these proteins. These dsRNA-associated proteins may have effects on how the cell senses and responds to v-dsRNA. Other modifications that could be influencing the cellular response to dsRNA that would survive extraction include methylation and 5′-tri- or diphosphates (14, 62). Mammalian reoviruses and poly I:C both have a 5′-diphosphate whereas ivt-dsRNA have 5′-triphosphate termini; both termini are able to activate RIG-I; however, the implication of this difference in rainbow trout is harder to elucidate due to the lack of RIG-I in this fish species (14, 63–65). Interestingly long ivt-dsRNA (>200 bp), which would lack a 5′-triphosphate was still able to activate RIG-I in murine embryonic fibroblasts (28). The cap status of the dsRNA molecules may also influence the host response; this has been best studied in terms of RIG-I and MDA5. The addition of an M7G cap partially reduced the RIG-I stimulatory properties of dsRNA, whereas 2′-*O*-methylation entirely abrogated RIG-I activation (66). In terms of MDA5, mutant viruses lacking 2′-*O*-methyltransferase induced higher MDA5-dependent type I IFN expression (67). In this study, v-dsRNA likely has a cap, as reoviruses put a 5′ cap on the positive strand of the genomic segments; in comparison, the *ivt*-dsRNA and poly I:C would not have a m7G cap (14). Kato et al. (28) found that capped *ivt*-dsRNA was still able to induce IFN-β production, unfortunately these molecules were not compared to uncapped molecules to determine if this had any positive or negative effects on stimulatory properties. Studies are currently underway to explore the role of different cap modifications on the host innate immune response.

Based on the assumption that ivt-dsRNA can act as a surrogate for v-dsRNA in inducing IFN at the cellular level, and the v-dsRNA vs. ivt-dsRNA comparison performed used molecules with the same sequence, it follows to test whether nucleotide sequence makes a difference. From the results of a group of three ivt-dsRNA containing host or viral sequences it appears as though sequence does not significantly affect ivt-dsRNA’s IFN inducing capabilities. This is congruent with the current literature that suggests dsRNA sequence is not a major influence on dsRNA receptor binding, as is length and structure (31, 58). Although there is evidence that RIG-I ligands generally have a uridine- or adenosine-rich ribonucleotide sequence, and OAS has a 4 bp-specific sequence motif, it is unclear if other receptors have any preference or sequence requirements, or whether there are any sequence preferences for fish dsRNA sensors (32, 68). Sequence may play a role in an antiviral state when RNAi is considered; however, in this system, there is an overwhelming IFN-mediated response that likely masks any RNAi effects (69).

Overall, this study sheds light on the dsRNA-mediated immune response in rainbow trout cells. The findings suggest that v-dsRNA produced by an aquatic reovirus is an IFN-inducing PAMP. There were no significant differences between a v-dsRNA molecule and length- and sequence-matched ivt-dsRNA molecules with regards to inducing IFN and ISGs and antiviral state, suggesting that ivt-dsRNA may be useful in studies of IFN and in future antiviral therapies for fish. This study sought to perform functional studies where possible, using antiviral assays to explore the biological relevancy of transcriptional quantification results. These findings contribute to a better understanding of the differences between dsRNA from different sources, which can help facilitate the production of more biologically relevant dsRNA-based therapies.

## Author Contributions

SP performed experiments and contributed to experimental design and writing of manuscript. SD-O contributed to experimental design, funding of project, and writing of manuscript.

## Conflict of Interest Statement

The authors declare that the research was conducted in the absence of any commercial or financial relationships that could be construed as a potential conflict of interest.

## Footnotes

^1^http://www.genomatix.de/cgi-bin/tools.

^2^www.graphpad.com.
